# Transcatheter Tricuspid Valve-in-Valve Procedure—An Illustrative Case Report and Review

**DOI:** 10.3390/jcm10174004

**Published:** 2021-09-04

**Authors:** Márcio José Montenegro da Costa, Edgard Freitas Quintella, Luiz Kohn, Maximiliano Otero Lacoste, Gustavo Lycurgo Leite, Leonardo Hadid, Dany David Kruczan, Ricardo Zajdenverg, Hugo de Castro Sabino, Paulo Antônio Marra da Motta

**Affiliations:** 1 Instituto Estadual de Cardiologia Aloysio de Castro, Rua Davi Campista, 326, Rio de Janeiro 22261-010, Brazil; e.quintella@gmail.com (E.F.Q.); leonardohadid@gmail.com (L.H.); Kruczan@centroin.com.br (D.D.K.); zajdenverg@globo.com (R.Z.); hc_sabino@terra.com.br (H.d.C.S.); 2 Hospital Universitário Pedro Ernesto, Boulevard 28 de Setembro, 77, Rio de Janeiro 20551-030, Brazil; luizkohn@hotmail.com; 3 Hospital Copa Star, Rua Figueiredo de Magalhães, 700, Rio de Janeiro 22031-012, Brazil; maxiolacoste@gmail.com; 4 Hospital Home, SGAS Quadra 613-Conjunto, Brasília 70200-730, Brazil; gustavolycurgo@gmail.com (G.L.L.); mottapa@uol.com.br (P.A.M.d.M.)

**Keywords:** tricuspid regurgitation, tricuspid valve, valve replacement, valve-in-valve

## Abstract

Severe tricuspid commitment is no longer understood as merely a marker of disease but is now widely thought of as a significant contributor to cardiac morbidity and mortality. However, isolated tricuspid valve surgery remains rare and to this day continues to be associated with the highest surgical risk among all valve procedures and high operative mortality rates, especially in reoperations. Therefore, the development of tricuspid transcatheter procedures is as necessary as it was for the other valves a couple of years ago. Recently, multiple percutaneous therapies have been developed for the management of severe tricuspid disease, initially only repair and more recently replacement, thus creating a new branch for the management of patients who have already undergone surgery and who present with dysfunctional bioprostheses. The purpose of this review and report is to demonstrate current and possible future challenges, and to show that the valve-in-valve procedure of the tricuspid valve is feasible and safe, and now can be performed in all its range, in the smallest to the largest sizes of presentation, without incurring the untoward risks of conventional surgery.

## 1. Introduction

The tricuspid valve (TV) is the largest and most apically positioned of the four cardiac valves [1]: its normal orifice area lies between 7 and 9 cm^2^ with an average gradient between the right atrium (RA) and right ventricular (RV) being typically <2 mm Hg [2]. Although TV is also an atrioventricular valve, its anatomy and function have several dissimilarities to the corresponding mitral valve (MV), in part due to the lower pressures in the right heart chambers.

The functional abnormalities resulting from TV disease are classified as primary and secondary, the first being relatively rare, and it is the consequence of a primitive lesion of the TV due to congenital or acquired disease processes that affect the leaflets or chordal structures, or both. Secondary TV disease is more common and is a consequence of other diseases such as left-side heart diseases, pulmonary hypertension, RV dilation, and dysfunction from any cause, without intrinsic lesion of the TV itself [1,3]. The most common TV disease in adults is tricuspid regurgitation (TR) and functional tricuspid regurgitation (FTR), with or without tricuspid leaflet tethering, secondary to left heart disease, either myocardial, valvular, or mixed, is responsible for more than 90% of TR in adults [3,4]. Irrespective of the specific initial etiology, TR is a progressive disease in the setting of RV and RA remodeling. Tricuspid stenosis (TS) is even more rarely described, accounting for about 2.4% of all cases of organic tricuspid valve disease, and most often coexists with mitral valve pathology, especially in patients with rheumatic heart disease [5].

TR is a common finding in most individuals, being found in up to 80–90% of them, and for long it was falsely considered a benign condition with a prolonged clinical latency [6]. Because of this, it has been relatively neglected both in the literature and clinically as compared to the primary left-sided diseases [3]. Furthermore, the rarity of TV surgical management, and its high post-operative mortality and morbidity [7,8,9], in addition to the lack of evidence proving the superiority of surgical treatment over medical therapy in severe TR [10], has led it to be called the “forgotten valve” for a long time [3,11].

Nonetheless, in the last few years, the pathophysiology and impact of TR on the outcome of various heart diseases has been increasingly understood and thus its non-benignity is already established in literature [12,13,14,15]. Long-term, higher FTR severity is associated with considerably worse survival, independently of other features [13], and despite TR providing no additive value in advanced congestive heart failure (CHF), it is associated with excess mortality in mild to moderate CHF [15]. Therefore, severe TR is associated with a poor prognosis, independent of age, biventricular systolic function, RV size, and dilation of the inferior vena cava [12].

Hence, a myriad of new research and surgical techniques has been developed aiming to establish the optimal treatment and to ensure perfect surgical timing [16]. Yet, to this day, TV surgery concomitant to left-sided heart surgery is the only Class I guideline recommended therapy for TR [17]. The remaining recommendations of the American and European societies lack both stronger evidence and clear benefits, whereas the current recommendation for isolated TV is restricted only to patients with severe TR who are either symptomatic or are developing progressive RV dilatation/dysfunction [17,18]. However, such patients with severe TR are often asymptomatic for a long period of time and symptoms may not specific, contributing to surgery’s delay [16].

Nonetheless, despite the renewed interest in earlier surgery for patients with severe isolated TR before the onset of severe RV dysfunction or end-organ damage and recent improvement in mid to long-term results [19,20,21], isolated TV surgery remains rare and to this day continues to be associated with the highest surgical risk among all valve procedures and high operative mortality rates, especially in reoperations [7,8,9]. In this framework, catheter-based therapies for patients with severe isolated TR has become an area of rapid evolution and growing interest [22], furthermore following the trend of valvular heart disease towards less invasive surgical and percutaneous therapies. As a result, multiple transcatheter tricuspid valve interventions (TTVI) have been developed for treating severe TR, at first aiming at TV repair and, more recently, transcatheter tricuspid valve replacement (TTVR) that allowed a new branch of possibilities in the management of TV disease. 

As the TV is being treated more often, also by surgical replacement, prostheses malfunctioning should be expected. Solutions as valve-in-valve and valve-in-ring with transcatheter aortic valve (TAVI) devices are in some way applicable to the tricuspid position. Some case reports have already been published and the most frequently used devices are the Melody™ (Medtronic, Minneapolis, MN, USA) transcatheter pulmonary valve (TPV) from Medtronic and the TAVI XT^®^ and SAPIEN 3^®^ (Edwards Lifesciences, Irvine, CA, USA) [23,24,25,26]. The first one has a major limitation as the maximum diameter size is 22 mm, and for the TV, it is too small. The procedures of valve-in-valve and valve-in-ring have been shown to be safe and feasible.

## 2. Imaging Assessment of the TV

### 2.1. Echocardiography

The goals of imaging in patients with TR are the assessment of severity, etiology, and consequences (RV size and function, pulmonary artery dimension, and pressure) and the detection of concomitant left-sided valvular disease (including the assessment of prosthetic valve function, where appropriate). In that regard, echocardiography remains the cornerstone imaging modality for initial assessment of the etiology and severity of TR. Clinically relevant measurements include an assessment of TR severity, tricuspid annular diameter, leaflet tenting measurements, and RV size and function [27].

Assessing the severity of TR by echocardiography remains challenging and controversial. Although the American Society of Echocardiography and the European Association of Cardiovascular Imaging guidelines considers three stages of FTR (mild, moderate, and severe) [28], the need has been felt for a more sensitive grading system [29]. A vena contracta ≥ 0.7 cm, effective regurgitant orifice area (EROA) of  ≥0.40 cm^2^, and regurgitant volume ≥ 45 mL, qualify it as severe; however, no distinction is made after these particular parameters. The TriValve registry showed that most of the TTVI patients have really severe TR (often called massive or torrential TR), and furthermore, it was noted that even after a significant TR improvement, in some cases it was still considered severe [30]. This is of particular importance for the evaluation of procedural results and residual TR, since it has been observed that a significant reduction in TR (although still severe according to the current guidelines) is associated with meaningful clinical benefits after TTVI [27]. Thus, a more sensitive five-stage TR severity assessment (mild, moderate, severe, massive, and torrential) has recently been proposed aiming to further assist the optimal therapy choice [31]. Massive and torrential TR gradings may have implications regarding selecting of patients eligible for percutaneous treatment [29]. Nevertheless, this new grading system is not yet present in the guidelines of the main international societies.

In the setting of TS, the echocardiographic view also provides the most useful information [18]. Echocardiographic evaluation of the anatomy of the valve and its subvalvular apparatus is important to assess valve reparability. The mean transvalvular pressure gradient is usually lower in TS than in mitral stenosis (MS), ranging between 2 and 10 mm Hg, and averaging around 5 mm Hg [5]. Despite no generally accepted grading of tricuspid stenosis severity existing, a mean gradient ≥ 5 mm Hg at normal heart rate and a calculated TV area of less than 1 cm^2^ is considered indicative of clinically significant TS [5,17,18]. Higher gradients may be seen with combined stenosis and regurgitation.

To this day, transthoracic echocardiography (TTE) and transesophageal echocardiography (TEE) represent the gold standard methods for preprocedural assessment of the TV [27], as well as tools for outcome prediction [4,32].

### 2.2. Cardiac Computed Tomography

Multislice computed tomography (CT) offers a comprehensive assessment of the real 3D anatomy, allowing the operator to assess the TV anatomy as well as the adjacent structures, including the proximity of the right coronary artery to the tricuspid annulus (TA), the coronary sinus, the hepatic veins, and vena cava [33]. Hence it has become the third-step imaging modality during pre-procedural planning for transcatheter TV interventions after TTE and TEE, as it provides valuable anatomic spatial information of the TV apparatus sometimes hampered by echocardiography because of its complex geometry and anterior position in the chest [19,22,34].

Its application is imperative when evaluating devices that directly interact with the TV leaflets or during the screening process for TTVR, as it enables the accurate measurement of the TA, including maximal anteroposterior and septolateral diameters, perimeter, area, and right ventricular geometry. It also enables the targeting of anchoring sites by drawing a perpendicular line linking the annular plane with the right ventricular septal free wall on a sagittal reconstruction [34]. Similar to transaortic (TAVR) and transmitral valve replacement (TMVR), preimplant CT plays an important role in defining fluoroscopic angles of coplanarity to the TA to help guide the procedure [35].

### 2.3. Cardiac Magnetic Resonance

Cardiac magnetic resonance (CMR) is currently the gold standard method for the assessment of RV morphology and function, besides an additive to 3D echocardiography for both anatomic and functional analysis of the TV and its annulus. Its use is particularly interesting for a comprehensive evaluation of the right-sided chamber without the need for radiation or iodinated contrast, and especially useful for those that require an appropriate three-dimensional assessment but have suboptimal echocardiographic windows (e.g., obese patients or with lung disease, breast implants, etc.) [28].

The measurement of TR severity on MRI might be performed using the indirect method by calculating TR volume, TR fraction, or by direct measurement of the effective regurgitant orifice area, when possible. While the TR severity cutoffs in CMR have not yet been established, a tricuspid regurgitant fraction ≥ 40% is generally considered hemodynamically significant [28].

## 3. Transcatheter Tricuspid Anatomic Challenges

Many lessons have been learned in the past decades about transcatheter valve replacement and although many of the concepts of TAVR and TMVR can be transposed to the TV [22], a better comprehension of the anatomical and functional peculiarities of the tricuspid valve and right heart chambers is essential to develop new techniques and to improve those already available, thereby overcoming the specific challenges related to TTVI.

First, surrounding the TV are four four key anatomic structures: the conduction system (atrioventricular node and right bundle of His), the right coronary artery, the noncoronary sinus of valsalva, and the coronary sinus ostium [1]. Therefore, the possible injury risks during transcatheter tricuspid interventions are elevated and with potential severe complications. Moreover, due to the trabeculated and thin RV wall, other approaches, such as the transapical, become hindering [22].

Compared with the MV, the tricuspid annulus is larger, with regurgitant orifice areas often twice those in the mitral position (up to 9 cm^2^ area in normal condition, much larger in the presence of functional TR); in addition, its leaflets are thinner and more fragile [22,27]. Hence, the major interventional issue related to the TV compared to the MV lies in its larger orifice. As such, a complete occlusion of the regurgitant area can be very troublesome with the current repair devices that were originally intended for smaller gaps. For the same reason, a replacement device would also have to be extremely large to cover and seal the entire TV area, especially in the absence of any type of annular calcification or leaflet—which is almost never seen in native tricuspid valves—to aid its anchoring [27].

The TA is a saddle-shaped ellipsoid that becomes planar and circular as it dilates [1,22]. Peculiarly, TA dilation does not occur symmetrically, occurring primarily in the anterolateral free wall in patients with left-sided heart disease with sinus rhythm, expanding mostly along the posterior border with less prominent leaflet tethering in patients with functional TR secondary to chronic atrial fibrillation (AF). Therefore, its preferential dilation of the anterior and posterior leaflets allows malcoaptation between the anteroposterior and posteroseptal commissures, rather than the anteroseptal commissure. This organic pattern has important therapeutic implications for TV repair, especially for leaflet-based approaches [22].

Similarly to the MV, the TV has a common antegrade approach. While the most used site is currently the transfemoral access, through the inferior vena cava (IVC), there are some devices that are delivered through a transjugular approach [22,31]. However, the absence of transseptal support and the short distance between the IVC orifice and the TV annulus, combined with the loss of anatomical landmarks under pathologic conditions (RA and RV dilation), makes catheter navigation even more cumbersome than in the setting of a mitral valve intervention, resulting in a complete lack of stabilization and difficult coaxiality, which can lead ultimately into an improper positioning of the repair/replacement device [22,31,32]. Besides the anatomical disadvantages, as tendency of cardiac implantable electrical devices (CIEDs) spreads, so will increase the difficulty of future TTVI. The presence of pacemakers, implantable cardioverter defibrillators (ICDs) or cardiac resynchronization therapy (CRT) devices will make navigating the catheter and fixing it in an optimal position even more problematic.

In addition to its anatomical peculiarities, the tricuspid valve also presents its own unique challenges on the imaging side. Since the TV is located more anteriorly compared to the MV, intraprocedural TEE guidance is particularly difficult in tricuspid procedures. In some circumstances, a combination of TEE, TTE, and intracardiac echocardiography (ICE) is needed to obtain adequate imaging quality [22].

Despite differences and challenges, in contrast to the mitral and aortic valves, the right heart valves are open-angulated and widely separated by the crista supraventricularis, making the risk of obstruction of the right ventricular outflow tract almost insignificant [22], thereby decreasing one of the most feared complications of percutaneous procedures.

## 4. Transcatheter Tricuspid Valve Repair or Replacement

There are several factors to consider when defining the repair or replacement strategy. Within the repair scope, most devices are designed to mimic surgical techniques, with the most used methods being: coaptation devices (Forma, ((Edwards Lifesciences, Irvine, CA, USA)), TriClip ((Abbott Vascular, Santa Clara, CA, USA)), Pascal ((Edwards Lifesciences)) direct suture annuloplasty (Trialign ((Mitralign, Tewksbury, MA, USA)), TriCinch (4Tech Cardio, Galway, Ireland) and ring annuloplasty devices (Cardioband ((Edwards Lifesciences)), Millipede IRIS ((Boston Scientific, Marlborough, MA, USA)). Currently, the off-label use of the MitraClip System (Abbott Vascular, Santa Clara, CA, USA) in TV position is the most widely used technique in the edge-to-edge repair and it has become the first-choice approach for high-risk patients with FTR, likely because of wide availability and operator familiarity [21,31]. Although there are still no specific guidelines and orientations on the subject, there are some key features that predict poorer results, and thus can be used as arguments against the use of the repair technique (Table 1).

One common exclusion for repair is the presence of a permanent ventricular pacing lead that interacts with the tricuspid leaflet, besides TS, which is prohibitive for any repair strategy because it will inevitably reduce valve area and increase the gradient. Certain congenital conditions such as Ebstein anomaly, as well as primary leaflet abnormalities due to endocarditis, inflammatory diseases, or iatrogenic causes, are also not suitable for percutaneous TV repair and would benefit more from a TTVR [36,37].

Severe leaflet tenting and very large tricuspid annular diameter are considered as predictors of inferior outcome in patients undergoing surgical tricuspid valve repair and therefore suggest that percutaneous repair will achieve similar subpoptimal results [36]. The presence of large coaptation gap > 6–8 mm and non-central regurgitant jets are also associated with poor procedural success [36]. Calcification in the potential grasping target and immobile or severely retracted leaflets (especially the septal leaflet) with extensive tenting distance are unlikely to have good outcomes with repair. If the predicted outcome will be inferior or if the residual tricuspid regurgitation is expected to be moderate or worse after repair, TTVR may be more appropriate.

Similarly to the repair strategy, there are also a few essential anatomic and geometrical features that can hinder an adequate TTVR (Table 2). Although there are larger valves in development, up to now, the largest valve in active trials in the U.S. is the 52 mm EVOQUE valve; therefore, those patients with a very large TA should be considered for a heterotopic caval valve implantation or a TV repair method [38,39]. Moreover, due to the larger size of the current delivery systems, the iliac veins, IVC, or RV may not be able to accommodate it. Other geometric factors such as the height, position, and angle of the IVC to the TA may make positioning of the transcatheter valve difficult or impossible [37]. Although there are still no long-term data, lifelong anticoagulation is generally recommended after TTVR. Therefore, in those patients with high bleeding a repair strategy may be preferable. Besides, transcatheter valves have the advantage that if needed in the future, valve-in-valve TTVR can be performed [37].

It is also worth pointing out that during the strategy choice, it should always be considered that in those patients with CHF, the complete elimination of TR may lead to afterload mismatch and worsening RV failure [36,37].

## 5. Transcatheter Tricuspid Valve Replacement

### 5.1. Orthotopic Valve Replacement

The first in-human transcatheter tricuspid valve-in-valve (TVIV) experience occurred in 2011 using an Edwards SAPIEN valve (Edwards Lifesciences, Irvine, CA, USA) [26,40]. Following that, a small number of reports of off-label use of transcatheter aortic and pulmonary valve prostheses for TVIV within dysfunctional surgical TV bioprostheses have been described [36]. Since then, the TVIV experience has expanded to include the Melody valve (Medtronic, Minneapolis, MN, USA) and newer-generation Edwards SAPIEN XT and SAPIEN 3 valves (Edwards Lifesciences, Irvine, CA, USA) [40], nowadays leading to a point where there are purpose-built devices in trials.

In general, in off-label application for surgical bioprosthesis with an outer diameter of ≤25 mm, the Melody valve (Medtronic, Minneapolis, MN, USA) may be preferable, while for a surgical bioprosthesis of ≥29 mm, SAPIEN (Edwards Lifesciences, Irvine, CA, USA)is preferred. Notwithstanding, there is room for dimensions between these depending on the results of balloon sizing during the procedure. The Melody valve (Medtronic) has an implant inner diameter of 22 mm (outer diameter 24 mm when implanted on a 22-mm delivery system), while the Edwards SAPIEN 3 valve (Edwards Lifesciences, Irvine, CA, USA) is available in sizes up to 29 mm, and its newer frame geometry with longer leaflets and a taller stent height allow overexpansion, with a resultant maximal diameter up to 31 mm with an additional 4 mL volume in the balloon [26].

The commonly described implantation routes are: transfemoral, transjugular, transatrial, and hybrid approaches using a thoracotomy. In general, for more vertically oriented tricuspid valves, the transfemoral approach is preferred to allow easier crossing and more coaxial deployment [40]. However, with the newer, more directable and flexible valve delivery systems, transfemoral delivery appears to be feasible in most cases because it allows one to overcome the acute angle between the inferior vena cava and the tricuspid valve. For extremely horizontal tricuspid valves, the transjugular approach, although not routinely used, may allow easier achievement of coaxiality, and it may also enable the procedure in cases with an occluded inferior vena cava.

In order to determine which valve is most appropriate for an individual, certain characteristics such as annular size, angle of the inferior vena cava, leaflet anatomy, and femoral access should be considered. One commonly encountered problem with severe functional TR is large annular size, therefore the Cardiovalve and EVOQUE valves are most likely to be successful due to their larger sizes. Cardiovalve, Intrepid, and EVOQUE have dedicated delivery systems with advanced steering, which may be necessary when anatomy is less than ideal; besides, Intrepid does not require leaflet capture in order to be deployed [37]. Considering the dimensions of the RV and venous system, in those patients with smaller anatomies, the EVOQUE has the advantage because of it small sheath 28-Fr size [38].

### 5.2. Heterotopic Valve Replacement

Despite recent achievements, there are still patients who are not eligible for TTVI, either due to geometric challenges that can lead to devices’ improper positioning or a low likelihood of beneficial outcome. Therefore, a therapeutic option beyond clinical treatment is necessary for those patients. In this framework, transcatheter caval valve implantation (CAVI) seems to be a feasible strategy to decrease systemic venous repercussions and improve right heart hemodynamics, thus relieving the effects of right heart failure [22,37,41].

The first in-human heterotopic transcatheter tricuspid valve implantation occurred in 2011 using a custom-made self-expanding heart valve that was implanted into the IVC [42]. Following that, reports have been made of performing CAVI as compassionate treatment in highly symptomatic patients with multiple advanced comorbidities, expanding the experience to bicaval approaches, leading to today where there are specific single caval protheses (TriCento ((New Valve Technology, Hechingen, Germany)) and bicaval prothesis (TricValve ((P+F Products + Features, Vienna, Austria)) [37,42].

Although feasibility and improvement of functional class has been proven [22,37,41,42], the heterotopic valve implantation approach should be considered a palliative procedure since it does not treat the TR itself nor its pathophysiology. The long-term impact of right atrial ventricularization is not yet known, but it is unlikely to have beneficial effects on ventricular function and RV remodeling. Furthermore, the procedure can be rather challenging due to the fact that it is performed in a region with a great volumetric variation, such as the IVC and the superior vena cava (SVC), which change according to the patient’s volumetric status, and the underlying risk of major complications, namely thromboembolism and hepatic vein occlusion [22,37,42].

## 6. Case Report

**Figure 1:**
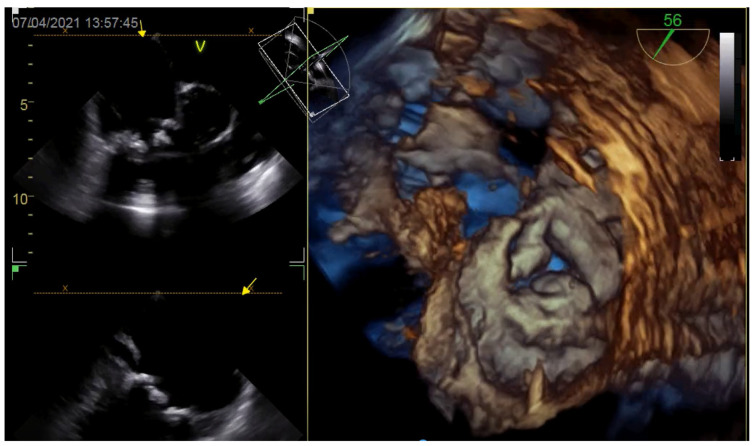
Three-dimensional transesophageal echocardiography showing the severely stenosed and regurgitant tricuspid bioprosthesis.

A 58-year-old female, with previous hypertension and diabetes, presented with progressive dyspnea, edema, and palpitations in NYHA class III. Twenty-seven years ago, she was submitted to a double valve replacement due to infective endocarditis with a mechanical prosthesis at the aortic position and a bioprosthetic one at the tricuspid position. In the in-hospital TEE, both, left and right ventricular systolic function and also the mechanical aortic prosthesis presented with no malfunctioning parameters. Heavy calcification and movement restriction of the tricuspid leaflets were characterized by severe TS and mild TR on the TEE (Figure 1). Maximum and medium tricuspid gradient measured 8.99 mm Hg and 5.97 mm Hg.

**Figure 2:**
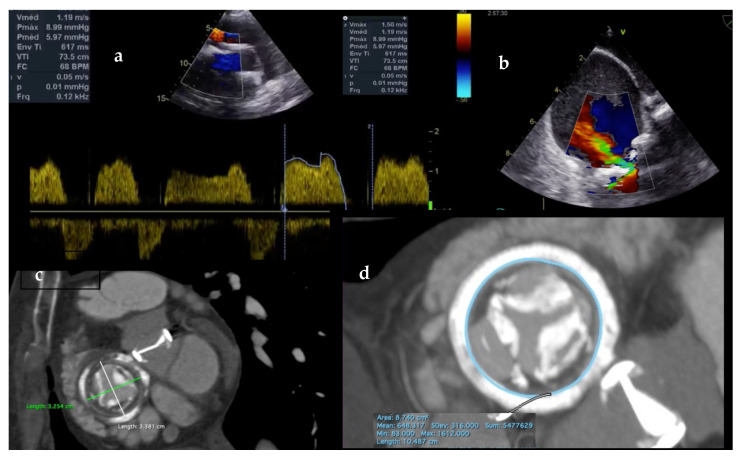
(**a**,**b**) Transesophageal echocardiography demonstrating RA–RV gradient and TV regurgitation. (**c**,**d**) Cardiac computed tomography showing bioprosthetic valve dimensions.

The indication and therapeutic approach were discussed by the multidisciplinary heart team. Despite a moderate STS Score = 5.17% and EuroScore II = 5.24%, a conventional open surgery was ruled out, considering the poor risk–benefit ratio expected as largely reported in the literature of isolated tricuspid reoperations [6,7,8]. TTVR was the therapy of choice for her. Angiografic computed tomography scans were acquired following the Aloysio de Castro State Institute of Cardiology protocol in Rio de Janeiro, Brazil. The analysis and the reconstruction of the TA was manually performed using the Horos software (LGPL 3.0; GNU Lesser General Public License, Version 3). The annulus estimated area was 874 mm^2^ and the perimeter of 106 mm, the prosthetic valve had an approximate 32 mm internal diameter (ID) and a 29 mm true ID was the manufacturer information (Figure 2).

### 6.1. Procedure

**Figure 3:**
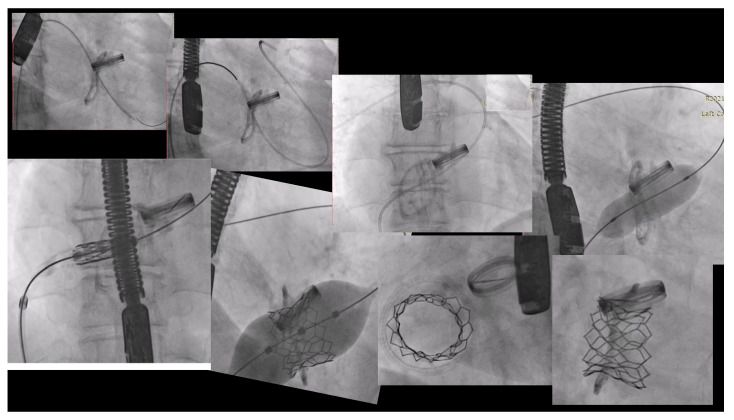
Tricuspid ViV procedure.

The interventional procedure was performed under general anesthesia and TEE guidance. No temporary pacemaker lead was implanted during the intervention. Right femoral vein access was gained, and an Python-14Fr™ (Meril Life Sciences Pvt. Ltd., Vapi, Gujarat, India) sheath was inserted. Transprosthetic valve access was achieved with a Hydrophilic Roadrunner JR 0.035 × 260 guidewire (Cook Medical, Bloomington, IN, USA) and anchored in the right pulmonary artery. It was then replaced by a Lunderquist DC 55 Guidewire (Cook Medical, Bloomington, IN, USA) which was also positioned in the distal right pulmonary artery branch. Predilatation of the bioprosthetic TV was done due to the tight valve area withholding of the transcatheter prosthetic valve using a 20 × 40 mm Mammoth—OTW Balloon Catheter (Meril Life Sciences Pvt. Ltd., Vapi, Gujarat, India). The delivery system was tracked over the wire and the valve was aligned with the previous bioprosthetic valve in a coaxial position. A MyVal TVH XL 32 was slowly deployed and anchored in the previous calcified leaflets and ring. Following valve implantation, the patient showed an immediate improvement in hemodynamics, maintaining circulatory stability. TEE revealed no significant TS (mean gradient 0.5 mm Hg) or hemodynamically significant regurgitation. The delivery system was removed, and the femoral vein access site manually compressed and a “8” shaped stitch was used to complete the hemostasis (Figure 3).

### 6.2. Clinical Course

The patient was immediately extubated still in the operating room and transferred to the intensive care unit. The patient was discharged two days later with no complications during her hospitalization. TTE at discharge showed a well expanded and fixed valve in optimal position, with good leaflet mobility and a mean gradient of 3.3 mm Hg, in addition to mild TR. The patient was followed clinically for 12 weeks, showing dramatic improvement of her congestive heart failure symptoms and improvement in functional class to NYHA I. No hemolysis has been observed.

## 7. Discussion

The illustrated report corroborates the literature demonstrating how it is feasible to perform such a procedure, even in patients with extensive TA, and the rapid improvement of functional class and quality of life that can be provided in those patients who were normally not considered for any invasive strategy.

The tricuspid valve-in-valve implantation is appealing for several technical reasons. The circular sewing ring of surgical bioprostheses enables the valve-in-valve sealing and offers a lower risk of perivalvular leak in comparison with transcatheter implantation in native valves. Furthermore, TV bioprosthetic are generally larger than those in the mitral or aortic position, achieving more favorable hemodynamics after TVIV [22,27,37].

Due to the relative immaturity of the field of TTVR, the full scope of complications is not yet fully known. In the same way, with no precise information about the previous planning there is a lack of information in how to choose the best prosthesis for the procedure. We used the manufacturer sizing information and the cardiac CT measures of the internal lumen of the previous surgically implanted bioprosthesis. Using the true lumen, we compared with the balloon expandable TAVI devices. As we found a diameter of 32 mm and an area of 874 mm^2^, the most safe choice was the 32 mm Meril, MyVal TAVI device. The implant was made with 7.5% of oversize with 3 mL over the nominal volume.

Another feature about this procedure is the recommendation to anchor the Lunderquist DC55 guide wire in the right branch of the pulmonary artery, as in this position it makes a straighter runway for the prosthesis. In this case there was such an immobility and rigidity in the surgically implanted valve that pre-dilation was mandatory. Even the balloon crossing the stenotic bioprosthesis was difficult. Finally, to achieve the best ViV positioning implant, we followed the metallic ring of the previous implanted TV till the best alignment, shown in Figure 3. The patient was kept in dual antiplatelet therapy (DAPT) for 30 days and kept in oral anticoagulation as needed due the aortic mechanical prosthesis.

As with other percutaneous strategies, anticoagulation remains a challenge. Lifelong anticoagulation has been recommended, though switching to dual antiplatelet agents after six months has been proposed when there is no concomitant indication for long term anticoagulation [37].

Despite recent progress, all patients undergoing TTVI are still patients with end-stage inoperable heart failure, RV dysfunction, severe comorbidities, and often as a compassionate treatment in many of the cases [22,27,30,32,37]. As such, there are still no specific guidelines and not enough evidence on the subject to support the selection of patients who might actually benefit from a percutaneous approach and those for whom it might be futile. Regardless of the current understanding of worsened cardiac outcomes related to TR, the debate whether patients with end-stage HF can benefit from interventional TV treatment is still valid. It has been shown that significant TR had no relevant survival impact in those patients with advanced HF, which may suggest that the correction or reduction of the regurgitant volume may not influence survival rates [13]. Therefore, accurate patient screening by a multidisciplinary heart team taking in to account LV and RV function, pulmonary artery pressure, comorbidities, frailty and life expectancy is of utmost importance to optimize results and effectiveness of TTVI.

## 8. Conclusions

In recent times, as the search for a better quality of life grows, TV malfunction has gained a new focus for transcatheter intervention, though much is still to be understood in this field. As the majority of patients being treated with these solutions are those with advanced dysfunctions, it becomes clear that the timing needs attention. Currently, TTVI has been shown to be feasible, safe, and capable of improving quality of life. Nevertheless, there are no appropriate physiological or anatomical benchmarks to guide better decisions. In stenotic cases, it is easier to find the best moment to indicate intervention, thus, the best technique is chosen in a tailored way. Meanwhile, there is a much wider field in the regurgitation landscape with numerous potential etiologies and hence a variety of pathophysiological aspects. In those FTR cases where RV dysfunctions lead to annular enlargement and valve tethering the preload is low, therefore the total correction of the regurgitation may be harmful. Although the rationale for the majority of cases is clear and its beneficial effects on the geometry and function of the right heart cavities are already notable, we still lack a thorough comprehension of the long-term physiological effects of TR correction, particularly its impact on the left heart cavities.

Patient selection is always paramount, and proper multimodality imaging with computed tomography, transthoracic and transesophageal echocardiography is crucial for deciding which procedure and which device is most appropriate for the individual patient. As the number of available devices increases, there will be more opportunities to treat those in need.

## Figures and Tables

**Table 1:** Potential contraindications to transcatheter tricuspid valve repair.

Potential Contraindications to Transcatheter Tricuspid Valve Repair
Primary valve abnormalities
Tricuspid stenosis
Permanent ventricular pacing lead that interacts with the tricuspid leaflet
Severe leaflet tenting and very large tricuspid annular diameter
Large coaptation gap > 6–8 mm and non-central regurgitant jets
Calcification in the potential grasping target and immobile or severely retracted leaflets (especially the septal leaflet)

**Table 2:** Potential contraindications to transcatheter tricuspid valve replacement.

Potential Contraindications to Transcatheter Tricuspid Valve Replacement
Large tricuspid annulus
Iliac veins, inferior vena cava or right ventricle not fitting delivery system
High bleeding risk
Right ventricle and tricuspid annulus incompatible geometry

## Data Availability

The authors confirm that the data supporting the findings of this study are available within the article.
